# Angle-Awareness Based Joint Cooperative Positioning and Warning for Intelligent Transportation Systems

**DOI:** 10.3390/s20205818

**Published:** 2020-10-15

**Authors:** Zhi Dong, Bobin Yao

**Affiliations:** 1 College of Transportation Engineering, Chang’an University, Xi’an 710064, China; zdong@chd.edu.cn; 2 College of Electronic and Control Engineering, Chang’an University, Xi’an 710064, China

**Keywords:** angle-of-departure, truncated signal subspace, distance based weighting, joint cooperative positioning and warning, intelligent transportation systems

## Abstract

In future intelligent vehicle-infrastructure cooperation frameworks, accurate self-positioning is an important prerequisite for better driving environment evaluation (e.g., traffic safety and traffic efficiency). We herein describe a joint cooperative positioning and warning (JCPW) system based on angle information. In this system, we first design the sequential task allocation of cooperative positioning (CP) warning and the related frame format of the positioning packet. With the cooperation of RSUs, multiple groups of the two-dimensional angle-of-departure (AOD) are estimated and then transformed into the vehicle’s positions. Considering the system computational efficiency, a novel AOD estimation algorithm based on a truncated signal subspace is proposed, which can avoid the eigen decomposition and exhaustive spectrum searching; and a distance based weighting strategy is also utilized to fuse multiple independent estimations. Numerical simulations prove that the proposed method can be a better alternative to achieve sub-lane level positioning if considering the accuracy and computational complexity.

## 1. Introduction

Advanced vehicular assistance systems have become an upward trend with great attention in both academic research and industrial application [1,2]. During the evolution process from traditional human based driving to smart assisted driving, even to autonomous driving, the signal processing techniques aiming at diverse perception data will play a critical role. Especially, strengthening the safety [3,4], no matter what level the autonomous driving of on-road vehicles is, shall become a principal task in the future. One of the vital issues is how to guarantee an accurate localization for each on-road vehicle with the at least sub-meter level requirement [5,6].

As we know, global navigation satellite systems (GNSS), e.g., the Global Positioning System (GPS) and BeiDou Navigation Satellite System (BDS), are widely used for current vehicular localization and navigation; but they cannot always satisfy the rigorous requirements of some location based services (LBS) [7,8,9]. The reasons involve the navigating signals being inevitably blocked or multipath transmission in urban environments, the vehicle motion, the satellites’ absence, etc. More seriously, the navigating signals can be recorded with malicious tampering and retransmitted to the vehicular terminals. Besides the improvement of GPS and BDS with some inertial navigation (utilizing some on-board kinematic sensors such as odometers, accelerometers, gyroscopes, etc.), the laser imaging detection and ranging (LIDAR) technique can achieve a simultaneous localization and mapping for intelligent vehicles, which is now accepted by more and more industries [10,11,12,13]. However, there still exist some problems that cannot be neglected if it is to be commercialized [14]. The first one is the cost; and the second one, also the most important one, is that the quality of LIDAR images will deteriorate because of the weak reflectivity of the wet road surface, which results in some detected region disappearing in the LIDAR images (such a difference between LIDAR images and map images will affect the further similarity calculation); in addition, the irregular snow lines inside the lane and near the roadsides also can confuse the lane identifiability. Both situations will bring great hidden danger to intelligent vehicles.

The wireless communication techniques provide a new alternative solution, which can become a powerful supplement to localization. For example, the vehicular ad-hoc networks (VANETs) have been designed with dedicated protocols and differentiated quality-of-service to achieve a connected road environment [15,16], including vehicle-to-vehicle (V2V) and vehicle-to-infrastructure (V2I) communications. In that sense, the cooperative positioning (CP) [17,18] becomes an effective measure to improve the localization accuracy by jointly fusing multiple location-related parameters exchanged among a series of VANET nodes. Generally, these location based parameters include maximum movable distance [19], channel state information (CSI) [20], received signal strength indicator (RSSI) [21,22], time-of-arrival (TOA), and time-difference-of-arrival (TDOA) [23,24].

In [25], two road-side units (RSUs) are utilized to broadcast their position and road geometry information, the vehicle combining them with odometer data and Doppler shift of the received signals to achieve a lane-level localization accuracy. References [26,27] investigated a vehicle-to-infrastructure based CP where each vehicle measures its position through the direction-of-arrival (acquired by a uniform linear array on the vehicle) and the known RSU position (acquired in each beacon packet). Such CP gains a better performance than GPS based localization. However, there exist four potential shortcomings. The first one is that the vehicle motion and the road unevenness will generate mechanical vibration so that the ULAbecomes unstable, which consequently influences DOA estimation; the second one is that the accuracy of DOA estimation highly depends on the array aperture so that the vehicle should be equipped with a larger scale antenna array, which will increase the cost. Although the coprime-array [28,29] can achieve increased degrees of freedom compared with the traditional uniform array, it still requires complicated operations; the third one is that one-dimensional angle estimation alone cannot distinguish the vehicle in the adjacent lane, which will induce the ambiguity of the spatial position; the last one is the angle estimation algorithm because it must simultaneously consider the estimation accuracy and computational complexity rather than focus on one of them [30], which is a basic requirement for timeliness. In addition, among the above CP strategies, an important function, i.e., the cooperative warning with respect to traffic safety, is neglected. In a highly connected road environment, each intelligent vehicle has the responsibility of guaranteeing the driving safety and road efficiency, that is the so-called cooperative safety. Such cooperation is embodied at least in an active reporting of the traffic accident or vehicle fault by V2I links to RSUs; consequently, each individual road, even the whole road networks, can gain global control.

In this paper, aiming at solving the aforementioned impasses, we introduce a novel joint cooperative positioning and warning system on the basis of spatial angle information. The positioning mainly depends on the wireless V2I links and kinematic model without considering the GNSS. The warning is depicted by the safety distance and the vehicle’s deceleration. Besides the detailed design of sequential allocation for CP warning tasks and the data format of localization packets, we also propose the computationally efficient AOD estimation algorithm and multiple detection fusing strategy to achieve sub-lane level positioning. To summarize, the main contributions of this paper are three-fold:

1. An angle-awareness based framework of the joint cooperative positioning and warning system is first discussed, which includes the positioning model based on state representation, the warning mechanism based on safety distance, and the CP warning task allocation and related data formats for periodic interaction.
2. To decrease the computational complexity, a truncated signal subspace based algorithm for angle estimation is proposed, which avoids matrix eigen decomposition and spectra searching.
3. To decrease the adverse influence of the near-far effect caused by angle estimation and improve the positioning accuracy, a distance based weighting strategy is also designed, which only utilizes the estimated positions without extra calculations.

The rest of this paper is organized as follows: In Section 2, the overview of the joint cooperative positioning and warning (JCPW) system is introduced. More details on the proposed algorithm are described in Section 3. Section 4 discusses the distance based weighting strategy for the initial position estimation. The numerical results are shown in Section 5, and Section 6 gives the conclusions.

Notation: ${\left(\cdot \right)}^{*}$, ${\left(\cdot \right)}^{T}$, and ${\left(\cdot \right)}^{H}$ denote the complex conjugate, transpose, and Hermitian transpose, respectively. Symbol “⊗” denotes the Kronecker product.

## 2. Joint Cooperative Positioning and Warning Overview

**Figure 1:**
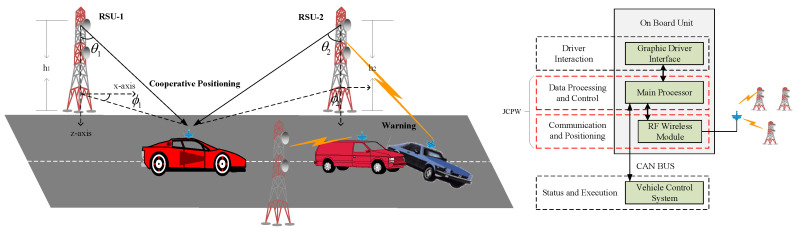
A graphical illustration of joint cooperative positioning and warning (JCPW).

We assume as a reference scenario in Figure 1 a fully connected intelligent vehicle network deployed along a given road segment with a double-lane width equal to *W* meters, belonging to an urban canyon environment. Without loss of generality, two different kinds of nodes are present: L RSUs, $\left\{R_{1},R_{2},\ldots ,R_{L}\right\}$, placed on the roadsides; and multiple intelligent vehicles, randomly located on the lanes, are traveling along arbitrary trajectories. The JCPW system mainly depends on the on-board processor and RF wireless module to achieve data transceiving and positioning.

### 2.1. Cooperative Positioning

For intelligent vehicle *V*, if requiring sub-lane-level localization accuracy, a more appropriate way is to perform self-positioning with the aid of multiple RSUs’ cooperation. There are two main reasons. One is that the centralized positioning schemes will give rise to a heavy computational load for RSUs, and they usually require complex algorithms or multi-user schemes to discern vehicles. The other is that, although the V2V communications can provide relative position information, it is usually unreliable because the communication links vary dynamically and are vulnerable to being blocked or having severe distortion [31]. Besides, GNSS is also a common choice. However, taking the GPS for example, it suffers from GPS signal blockage and multipath, as well as inadequate accuracy (∼10 m) [32]. Therefore, we only consider the V2I communications, in which the line-of-sight (LOS) path generally dominates in such a condition [33]. In addition, the cooperative positioning in our system refers to multiple RSUs’ cooperation for achieving decentralized positioning.

The CP stage includes the localization data transmitting and receiving, AOD estimating, and position calculating. The first event is in time span $T_{TR}$, and the last two events are in time span $T_{P}$, that is to say, a CP interval $T_{CP}=T_{TR}+T_{P}$. For a better understanding, let the RSU position $p_{R}={\left[x_{R},y_{R},z_{R}\right]}^{T}$ be fixed and exactly known, while the vehicle’s position $p_{V}\left(t\right)={\left[x_{V}\left(t\right),y_{V}\left(t\right),z_{V}\left(t\right)\right]}^{T}$ is contrary. It is reasonable to consider that the velocity of the vehicle keeps nearly invariant during $T_{TR}$, and the acceleration contributes very little due to the sufficiently high packet rate. The real-time velocity reading $v\left(t\right)={\left[v_{x}\left(t\right),v_{y}\left(t\right)\right]}^{T}$ and acceleration reading $a_{V}\left(t\right)={\left[a_{x}\left(t\right),a_{y}\left(t\right)\right]}^{T}$ can be acquired from the vehicular sensors. Besides, the two-dimensional AOD information θ and ϕ denotes the elevation (i.e., the angle between the z-axis and the LOS signal) and azimuth (i.e., the angle between the x-axis and the projection of the LOS signal) of a vehicle, respectively, which is defined by the Cartesian coordinates of the RSU; see Figure 1.

We define the vehicle’s state vector as:

(1) $$c\left(t\right)={\left[p_{V}^{T}\left(t\right)\;v^{T}\left(t\right)\right]}^{T}.$$

then in the *k*-th CP interval, k=1,2,…, the kinematics used in the positioning stage is a constant model with invariant velocity and acceleration. It is given by the following iterative formulas,

(2) $$c\left(t_{s}\right)=\left\{\begin{array}{cc} c\left(T^{k-1}\right)+w\left(t_{s}\right)\;\;\; & \mathrm{if}\;\;T^{k-1}\leq t_{s}<T^{k-1}+T_{TR}\\ \Gamma c\left(t_{s-1}\right)+\Xi a_{V}\left(t_{s-1}\right) & \mathrm{if}\;\;T^{k-1}+T_{TR}\leq t_{s}<T^{k} \end{array}\right.$$

where we use subscript “*s*” for discrete time indexes, $w(t_{s})$ represents the noise item, and:

$$\Gamma =\left[\begin{array}{ccc} I_{2} & 0_{2\times 1} & \left(t_{s}-t_{s-1}\right)I_{2}\\ 0_{1\times 2} & 1 & 0_{1\times 2}\\ 0_{2} & 0_{2\times 1} & I_{2} \end{array}\right],\;\;\;\;\;\Xi =\left[\begin{array}{c} \frac{1}{2}{\left(t_{s}-t_{s-1}\right)}^{2}I_{2}\\ 0_{1\times 2}\\ \left(t_{s}-t_{s-1}\right)I_{2} \end{array}\right]$$

where Γ denotes the state transition matrix that applies the effect of the vehicle’s state at time $t_{s-1}$ on the one at time $t_{s}$; and Ξ is the control matrix that applies the effect of acceleration $a_{V}\left(t_{s-1}\right)$ on the current vehicle’s state vector.

In (2), the first formula gives the initial position states, and the second one gives the estimations of subsequent positions for the left time span $T-T_{TR}$ with the help of velocity and acceleration readings. The elements $x_{V}\left(T^{k-1}\right)$ and $y_{V}\left(T^{k-1}\right)$ of $c(T^{k-1})$ are the only unknown parameters. Once the elevation θ and azimuth ϕ are estimated, which will be introduced in Section 3, we can further estimate the vehicle’s initial positions at time $T^{k-1}$ according to basic geometry relations, i.e.,

(3) $$\begin{array}{ccc} {\hat{x}}_{V}\left(T^{k-1}\right) & = & \frac{1}{K}\sum_{i=1}^{K}\omega_{i}\left[{\bar{z}}_{i}\tan{\hat{\theta }}_{i}\cos{\hat{\phi }}_{i}+x_{R_{i}}\right] \end{array}$$

(4) $$\begin{array}{ccc} {\hat{y}}_{V}\left(T^{k-1}\right) & = & \frac{1}{K}\sum_{i=1}^{K}\omega_{i}\left[{\bar{z}}_{i}\tan{\hat{\theta }}_{i}\sin{\hat{\phi }}_{i}+y_{R_{i}}\right] \end{array}$$

where ${\bar{z}}_{i}=z_{R_{i}}-z_{V}$. Herein, we consider K RSUs for cooperation. The weighting coefficients ${\left\{\omega_{i}\right\}}_{i=1}^{K}$ need to be designed to improve the accuracy of localization, which will be reserved for Section 4. Based on the above kinematic model and estimated position information, the whole trajectory would be retrieved.

It is worth mentioning that, based on (2), the positioning at time $T^{k}$ can be given by state vector $c\left(T^{k}\right)=\Gamma c\left(T^{k-1}\right)+\Xi a_{V}\left(T^{k-1}\right)$ and measurement vector $\gamma \left(T^{k}\right)=H(c\left(T^{k}\right))+v\left(T^{k}\right)$, where $H(c\left(T^{k}\right))$ is a nonlinear mapping function with respect to the AOD estimation and $v(T^{k})$ is noise. To reduce the influence of noise, the extended Kalman filter (EKF) [34] will be a better choice due to it being able to provide optimal position estimations in the mean-squared sense for a Gaussian noise distribution. However, two basic restrictions should be considered beforehand, i.e., the identification of the probability distribution and the determination of the covariance information of the measurement error, which are not easy tasks due to the limited number of sampling in actual scenarios. Therefore, we leave them as future works.

### 2.2. Warning

In our considered system, the traffic safety is guaranteed in an active manner, i.e., each intelligent vehicle periodically monitors the surrounding traffic status that is broadcast by RSUs; meanwhile, its own states, such as traffic accident, component fault, velocity, acceleration/deceleration rate, remaining energy, etc., should be reported in a timely manner. To do so, the RSUs can infer the global traffic status, and each vehicle can also acquire safety-related traffic parameters, for example deceleration and safety distance.

**Figure 2:**
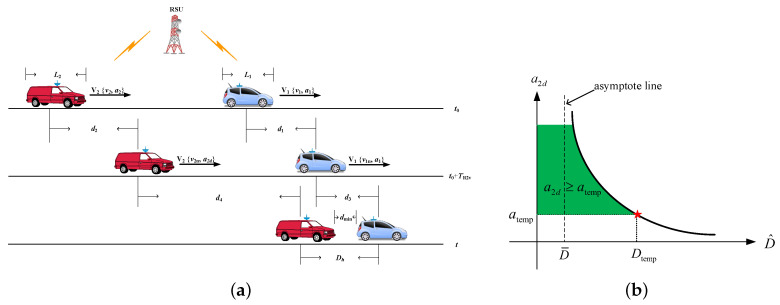
The warning systems: (**a**) two vehicle scenario; (**b**) a qualitative deceleration-distance curve.

We now consider a basic scenario that is depicted in Figure 2a, in which vehicle $V_{1}$ with length $L_{1}$, velocity $v_{1}$, and acceleration $a_{1}$ and vehicle $V_{2}$ with length $L_{2}$, velocity $v_{1}$, and acceleration $a_{1}$ are driving along a road. According to the vehicle kinematics, we can calculate the safety distance $D_{s}$ to characterize the warning strategy. The total safety distance includes three components. The first one is distance $D_{w}$, which represents the moving distance of vehicle $V_{2}$ after it receives the warning message from the RSUs and begins to decelerate. The second one is distance $D_{r}$, which represents the relative moving distance when $V_{2}$ slows down with deceleration $a_{d}$ until the relative speed between two vehicles becomes zero. The last one is the minimum headway distance $D_{h}$, which needs to be guaranteed when the relative speed reaches zero. Therefore, the safety distance is represented by:

(5) $$D_{s}=D_{w}+D_{r}+D_{h}$$

where $D_{w}=d_{2}-d_{1}$ and $d_{1}$ and $d_{2}$ denote the moving distance of $V_{1}$ and $V_{2}$ in time span $T_{R2v}$, respectively, so that $D_{w}=\left(v_{2}-v_{1}\right)T_{R2v}+\frac{1}{2}\left(a_{2}-a_{1}\right)T_{R2v}^{2}$. As we know, the velocity difference of two vehicles at time $\left(t_{0}+T_{R2v}\right)$ is $\Delta v=v_{2n}-v_{1n}=\left(v_{2}-v_{1}\right)+\left(a_{2}-a_{1}\right)T_{R2v}$, and vehicle $V_{2}$ begins to slow down with a desired deceleration $a_{2d}$, which will generate relative deceleration $\Delta a=a_{2d}-a_{1}$; therefore, we can get $D_{r}=d_{4}-d_{3}=\int_{0}^{\frac{\Delta v}{\Delta a}}\left(\Delta v-\Delta a\cdot t\right)dt=\frac{{\left(\Delta v\right)}^{2}}{2\Delta a}$. The minimum headway distance $D_{h}=\frac{1}{2}\left(L_{1}+L_{2}\right)+d_{min}$, where $d_{min}$ is the minimum distance between two vehicles.

It is worth mentioning that the desired deceleration $a_{2d}$ in Equation (5) is the only unknown parameter, which has a direct relationship with the warning strategy. By simple derivation, the estimated desired deceleration:

(6) $${\hat{a}}_{2d}=\frac{{\left(\Delta v\right)}^{2}}{2(\hat{D}-D_{w}-D_{h})}+a_{1}$$

where $\hat{D}$ is the estimated inter-vehicle distance *D* based on the vehicles’ positions. The qualitative deceleration-distance curve is shown in Figure 2b. For a given distance $D_{\mathrm{temp}}$, the corresponding $a_{\mathrm{temp}}$ is the least desired deceleration. Vehicle $V_{2}$ performs a reasonable braking according to the real-time distance information for avoiding accidents.

**Remark 1.**  *For the above qualitative analysis, it actually relies on some assumptions, i.e., vehicle $V_{1}$ keeps its constant deceleration before stopping; vehicle $V_{2}$ keeps its acceleration unchanged during $T_{R2v}$ and then slows down with an assigned constant deceleration no less than that of vehicle $V_{1}$. More complicated scenarios and related parameters’ evaluation will be left for future work. Given that the measured distance $\hat{D}$ depends on the position of two vehicles, hence the following work will focus on the problem of the vehicle’s positioning.*

### 2.3. Task Sequence and Data Frame Format

**Figure 3:**
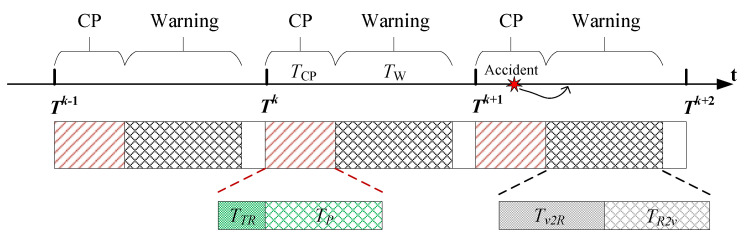
A graphical representation of the task sequences. CP, cooperative positioning.

The JCPW system refers to two important sub-functions: positioning and warning; therefore, an efficient task sequence should be designed. We first give a detailed explanation and analysis about the designed task sequences. For convenience, the basic structure is shown in Figure 3.

**Figure 4:**
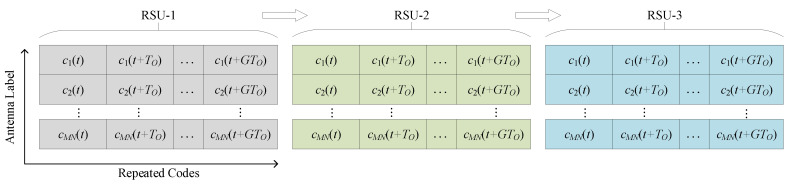
A graphical illustration of the localization data format.

For one time interval *T*, there are three parts. The first one with time interval $T_{\mathrm{CP}}$ is used for positioning. Without loss of generality, we assume K RSUs participate in the current CP. That is to say, $KT_{L}$ is required, where $T_{L}$ denotes the allocated time span of localization data for one RSU. In each $T_{L}$, a series of orthogonal code sequences c(t) with length $T_{O}$ is transmitted to assist the vehicle in AOD detection. The data format is shown in Figure 4, where we depict a case that K=3 RSUs, and the orthogonal sequence c(t) is repeated G+1 times with a total length $T_{L}=\left(G+1\right)T_{O}$.

As shown in the aforementioned kinematic models (see Equation (2)), during the AOD detections, all the data packets transmitting from RSUs and received by the vehicle are completed in a time span of $T_{TR}$. Due to the speed of light being much larger greater the vehicle’s speed and the high data rate of the orthogonal sequences, it is reasonable to neglect the time consumption in the stage of transmitting and receiving; consequently, a computationally efficient algorithm for AOD detection is very necessary for accurate localization. That is to say, the time span of data processing, i.e., $T_{P}=T_{CP}-T_{TR}$, will become a dominant factor in the subsequent localization. In $T_{TR}$, the vehicle’s position is roughly thought to be kept unchanged; after that, it will be calculated by inertial equations. Obviously, a small $T_{P}$ is a benefit for reducing the position error. In addition, during the data processing, multiple two-dimensional AODs are estimated separately due to the RSUs cooperating in a successive manner. Strictly speaking, this AOD information cannot be transformed into the same position because the vehicle is moving in $T_{TR}$; however, the time differences between the successively transmitted localization data from different RSUs are also negligible; that is to say, we can consider all K estimated AODs as effective measurements for the initial position $x_{V}\left(T^{k-1}\right)$ and $y_{V}\left(T^{k-1}\right)$. For example, consider such a scenario in which all K=5 RSUs locate approximately 100 m around the vehicle; the system bandwidth is 10 MHz; the localization data codes are selected from a 64×64 Hadamard matrix, and each repeat 20 times; the vehicle moves 20 m/s along the longitudinal direction. Only considering the line-of-sight communication link, we can see that, during one localization data time, the vehicle moves forward 0.256 cm; and all five RSUs will contribute a movement of 1.28 cm.

The second part with time span $T_{W}=T_{v2R}+T_{R2v}$ is used for warning. In this stage, there are two basic contents, i.e., the active state information reporting from the vehicle (to the RSUs) with time span $T_{v2R}$ and the periodic traffic status received from the RSUs (to the vehicle) with time span $T_{R2v}$. In the time sequence, the first event has the highest priority because all other vehicles have to depend on the current vehicle’s status to make a reasonable strategy for further driving. Different from the common state information, those emergent events, for example traffic accidents, loosing control, malfunction, etc., are much more important for traffic safety. In particular, given the randomness of traffic accidents, the conflict of the tasks in the time sequence is inevitable; see Figure 3, in which the accident labeled by a red star occurs in the CP stage. In such scenarios, the vehicle’s on-board central processing unit should execute an interruption and switch to the warning stage. If the active reporting module still works, the last time positioning results and other related vehicle state information will automatically report to the RSUs; if it unfortunately falls into a breakdown, the subsequent vehicles will take responsibility for uploading the accident information. By doing so, the system can be guaranteed a timely traffic status reporting.

Besides the above two important parts, the third one is reserved for further exploiting functions, for example vehicle platoon control, high definition map matching, and so on. The reserved time span is $T-T_{CP}-T_{W}$.

## 3. Computationally Efficient Two-Dimensional AOD Estimation

Different from the research works [27,35], we focus on the computationally efficient AOD estimation rather than simply assuming they are obtained beforehand or utilizing directional antennas. We herein just take one RSU for example in the following content because the others share the same data model and processing procedures.

### 3.1. Data Model

In the designed CP system, the RSU is equipped with a uniform rectangular array (URA) with M×N omnidirectional antenna elements, and the vehicle is equipped with a single omnidirectional antenna. The Cartesian coordinate is shown in Figure 1. Let the antenna element in origin *o* be the reference point. The (m,n)-th antenna locates in the xoy plane with coordinate $\left(\frac{\lambda }{2}m,\frac{\lambda }{2}n,0\right)$, where λ is the wavelength of the carrier frequency, antenna element spacing d=λ/2, and m=0,1,…,M−1; n=0,1,…,N−1.

We assume that the time synchronization and frequency synchronization between the RSU and vehicle have been calibrated. The V2I communication is in the line-of-sight (LOS) condition, which is depicted by the Rician channel model with parameter κ [36]. In addition, the system is working in narrow-band. Based on the designed localization data frames, all MN antennas simultaneously transmit orthogonal code sequences $c_{q}\left(t\right)$, q=1,…,MN. The signals propagate from the RSU to the vehicle over different paths, resulting in the superposition of multipath components (MPCs); therefore, besides the LOS signal component, it is reasonable to assume that there are *K* MPCs, each of which has its own AOD pairs $\theta_{p}$ and $\phi_{p},p=1,2,\ldots ,K$. Let the propagation delay $\tau_{p}$, attenuation coefficient $A_{p}$, and AOD pair $\left\{\theta_{p},\phi_{p}\right\}$ denote the *p*-th parameterized path [33,37]. Without loss of generality, given that the wireless environment has to undergo changes due to the vehicle’s movement, we assume the attenuation coefficient $A_{p}$ is block-variant, i.e., it remains unchanged within $T_{O}$, but varies with different $T_{O}$; the LOS signal component is deemed as the first path in the following derivation.

Therefore, the complex baseband signal received by the vehicle can be expressed as [37,38]:

(7) $$\begin{array}{c} r\left(t\right)=\sum_{p=1}^{K}\sum_{q=1}^{MN}\sum_{g=0}^{G}A_{p}\left[g\right]e^{j\varphi_{q}\left(\theta_{p},\phi_{p}\right)}c_{q}\left(t-gT_{O}-\tau_{p}\right)+n\left(t\right) \end{array}$$

where *g* indexes the transmission of $c_{q}\left(t\right)$; n(t) is zero-mean Gaussian white noise with covariance $\sigma_{n}^{2}$. For the *p*-th signal component, $\varphi_{q}\left(\theta_{p},\phi_{p}\right)$ represents the phase difference between the *q*-th antenna and the reference one, obviously, $\varphi_{1}\left(\theta_{p},\phi_{p}\right)=0$.

We herein neglect the delay difference between MPCs, i.e., $\tau_{1}\approx {\left\{\tau_{p}\right\}}_{k=2}^{P}$, and $\tau_{1}$ induced by the LOS component can be effectively eliminated by correlation detection. By performing match-filtering, we can get:

(8) $$y_{m,n}\left[g\right]\approx \sum_{k=1}^{P}A_{p}\left[g\right]e^{j\left(m-1\right)\mu_{k}}e^{j\left(n-1\right)\upsilon_{k}}+w_{m,n}\left[g\right]$$

where:

(9) $$\mu_{k}=\frac{2\pi d\sin\theta_{k}\cos\phi_{k}}{\lambda },\;\;\upsilon_{k}=\frac{2\pi d\sin\theta_{k}\sin\phi_{k}}{\lambda }$$

$y_{m,n}\left[g\right]$ is the match-filtered result with respect to the {m,n}-th antenna in the URA, and $w_{m,n}\left[g\right]$ is the noise after match-filtering.

Let $a(\mu_{k})$ and $b(\upsilon_{k})$ be the *x*-axis and *y*-axis steering vectors,

(10) $$\begin{array}{ccc} a(\mu_{k}) & = & {\left[1,e^{j\pi \mu_{k}},e^{j2\pi \mu_{k}},\ldots ,e^{j\pi \left(M-1\right)\mu_{k}}\right]}^{T} \end{array}$$

(11) $$\begin{array}{ccc} b(\upsilon_{k}) & = & {\left[1,e^{j\pi \upsilon_{k}},e^{j2\pi \upsilon_{k}},\ldots ,e^{j\pi \left(N-1\right)\upsilon_{k}}\right]}^{T} \end{array}$$

and let:

(12) $$y\left[g\right]={\left[y_{1,1}\left[g\right],y_{2,1}\left[g\right],\ldots ,y_{M,1}\left[g\right],\cdots ,y_{M,N}\left[g\right]\right]}^{T}$$

then it has:

(13) $$y\left[g\right]=\left[b\left(\upsilon_{1}\right)⊗a\left(\mu_{1}\right),\ldots ,b\left(\upsilon_{K}\right)⊗a\left(\mu_{K}\right)\right]s\left[g\right]+w\left[g\right]$$

where vector $s\left[g\right]={\left[A_{1}\left[g\right],A_{2}\left[g\right],\ldots ,A_{K}\left[g\right]\right]}^{T}\in C^{K\times 1}$, and noise vector w[g] has the same form as y[g]. Furthermore, defining $A=\left[a\left(\mu_{1}\right),a\left(\mu_{2}\right),\ldots ,a\left(\mu_{K}\right)\right]\in C^{M\times K}$, $B=\left[b\left(\upsilon_{1}\right),b\left(\upsilon_{2}\right),\ldots ,b\left(\upsilon_{K}\right)\right]\in C^{N\times K}$, therefore, the joint array manifold $\tilde{A}=B⊙A$, so Equation (13) can be expressed by:

(14) $$y\left[g\right]=\tilde{A}s\left[g\right]+w\left[g\right]$$

### 3.2. Truncated Signal Subspace for AOD Estimation

Many research works such as [26,27] directly utilize the multiple signal classification (MUSIC) algorithm to achieve angle estimation. The basic spectrum function is given by:

(15) $${P\left(\theta ,\phi \right)|}_{\left(\theta ,\phi )\Leftrightarrow (\upsilon ,\mu \right)}=\frac{1}{{\left[b(\upsilon )⊗a(\mu )\right]}^{H}U_{0}U_{0}^{H}\left[b(\upsilon )⊗a(\mu )\right]}.$$

where the noise subspace $U_{0}$ is acquired by the eigenvalue decomposition of $R=E\{y\left[g\right]y^{H}\left[g\right]\}$, i.e.,

(16) $$R=\left[U_{S}\;U_{0}\right]\left[\begin{array}{cc} \Sigma_{S} & 0\\ 0 & \Sigma_{0} \end{array}\right]\left[\begin{array}{c} U_{S}^{H}\\ U_{0}^{H} \end{array}\right]$$

where signal subspace $U_{S}\in C^{MN\times K}$ consists of the eigen-vectors with respect to the *K* largest eigenvalues (that construct $\Sigma_{S}=\mathrm{diag}\left\{\eta_{1},\eta_{2},\ldots ,\eta_{K}\right\}$), and the remnant eigen-vectors form noise subspace $U_{0}$.

However, there are some restrictions for P(θ,ϕ) in actual scenarios. First, it is difficult to determine the real number of MPCs, which will result in the erroneous partition of the signal subspace and noise subspace. Specifically, if the MPCs are coherent with each other, the MUSIC algorithm has to solve the rank-deficient problem at the cost of decreasing the array aperture. Second, the globe searching in the two-dimensional angle domain is very exhaustive, which will consume huge time complexity. In addition, the identification of the LOS component also requires extra calculations.

As shown in the aforementioned task sequences, the time complexity of the parameter estimation during CP processing has a direct influence on the positioning error. That is to say, the two-dimensional AOD estimation algorithm for the LOS signal component should simultaneously satisfy the requirements for lower computational complexity and higher estimation accuracy. To achieve such an objective, we mainly adopt two techniques.

On the one hand, in order to effectively utilize the observed data Y=[y[0],y[1],…,y[G]], we can double the length of the data by taking advantage of the complex conjugate version, i.e., constructing the so-called forward-backward observing matrix [39],

(17) $$\tilde{Y}=\left[Y\;\;JY^{*}\right]$$

where J is the matrix with ones on its anti-diagonal and zeros elsewhere. Such an operation can promote the accuracy of AOD estimation.

On the other hand, we try to directly extract the signal subspace with respect to the LOS signal without performing matrix decomposition. According to the subspace theory, $U_{S}$ and $\tilde{A}$ span the same signal subspace, namely $U_{S}=\tilde{A}T$, where T is a nonsingular matrix. In our scenario, the LOS signal component plays a dominant role in the propagation environment, i.e., it usually has the strongest power; therefore, the truncated signal subspace $u_{1}\in C^{MN\times 1}$, which is defined by the eigenvector corresponding to the largest eigenvalue, is actually a low-dimensional approximation to the signal subspace $U_{S}$. Strictly speaking, the subspace dimension reducing indeed results in partial loss of MPC information; however, we are only concerned with the LOS signal component because it is directly related to the vehicle’s position. As we know, the depiction of the dominant LOS signal is the Rician κ factor, which is defined by the power ratio between the LOS component and other MPCs. Larger κ means the array steering vectors of the LOS component contribute more in the signal subspace; hence, we can use the truncated signal subspace $u_{1}$ as an estimation to the array manifold vector ${\tilde{a}}_{LOS}$, i.e., $u_{1}\approx \tau_{1}{\tilde{a}}_{LOS}$, where $\tau_{1}$ denotes the value in the first row and first column of T.

To this end, we introduce an iterative method, which is based on the power iteration scheme [40]. The basic principle is as follows. Considering an initial non-zero vector $u_{t}^{\left(0\right)}\in C^{MN\times 1}$, it can be expressed in terms of the linear combination of all eigen-vectors of R, i.e.,

(18) $$u_{t}^{\left(0\right)}=\alpha_{1}u_{1}+\sum_{k=2}^{K}\alpha_{k}u_{k}$$

Left-multiplying both sides of (18) by the covariance matrix R, we have:

(19) $$Ru_{t}^{\left(0\right)}=\alpha_{1}Ru_{1}+\sum_{k=2}^{K}\alpha_{k}Ru_{k}=\alpha_{1}\eta_{1}u_{1}+\sum_{k=2}^{K}\alpha_{k}\eta_{k}u_{k}.$$

If we repeat the above multiplication *l* times, i.e.,

(20) $$R^{l}u_{t}^{\left(0\right)}=\alpha_{1}\eta_{1}^{l}u_{1}+\sum_{k=2}^{K}\alpha_{k}\eta_{k}^{l}u_{k}\;⟺\;\frac{R^{l}u_{t}^{\left(0\right)}}{\eta_{1}^{l}}=\alpha_{1}u_{1}+\sum_{k=2}^{K}\alpha_{k}\frac{\eta_{k}^{l}}{\eta_{1}^{l}}u_{k}.$$

As we can see, due to the LOS signal being dominant compared with other MPCs in our considered scenario, i.e., $\eta_{1}>\eta_{2},\eta_{3},\ldots ,\eta_{K}$, hence the following conclusion holds when l→∞,

(21) $$u_{t}≜\frac{R^{l}u_{t}^{\left(0\right)}}{\eta_{1}^{l}}\to \alpha_{1}u_{1}$$

The conclusion in Equation (21) illustrates that $u_{t}$ is a reasonable approximation of the truncated signal subspace $u_{1}$. Based on that, we summarize the above procedures in Algorithm 1.
**Algorithm 1** Truncated signal subspace extraction.**Input:** The estimated auto-correlation matrix $\hat{R}=\tilde{Y}{\tilde{Y}}^{H}\in C^{MN\times MN}$;**Output:** The estimated ${\tilde{a}}_{LOS}$;
1:Initializing2: l=1; $\epsilon =10^{-3}$3: Auxiliary vectors $u_{t}^{\left(0\right)}={\left[1,0,\ldots ,0\right]}^{T}$ and $u_{t}^{\left(1\right)}={\left[1,1,\ldots ,1\right]}^{T}$;4:**while**$\|u_{t}^{\left(l\right)}-u_{t}^{\left(l-1\right)}\|\geq \epsilon$**do**5: l=l+1;6: $u_{t}^{\left(l\right)}=Ru_{t}^{\left(l-1\right)}$;7:**end while**8:**return**$u_{t}=u_{t}^{\left(l\right)}$.

Once we get $u_{t}$ by Algorithm 1, according to the following approximate relation,

(22) $${\tilde{a}}_{LOS}\approx \gamma u_{t}$$

where γ is an unknown complex constant. We can directly calculate the two-dimensional AOD of the LOS signal component, i.e.,

(23) $$\begin{array}{ccc} {\hat{\mu }}_{1} & = & \frac{1}{M(N-1)}\sum_{i=1}^{M}\sum_{k=2}^{N}∠\left[\frac{{\tilde{a}}_{LOS}\left(i,k\right)}{{\tilde{a}}_{LOS}\left(i,k-1\right)}\right] \end{array}$$

(24) $$\begin{array}{ccc} {\hat{\upsilon }}_{1} & = & \frac{1}{N(M-1)}\sum_{k=1}^{N}\sum_{i=2}^{M}∠\left[\frac{{\tilde{a}}_{LOS}\left(i,k\right)}{{\tilde{a}}_{LOS}\left(i-1,k\right)}\right] \end{array}$$

where ∠[·] is to get the phase. ${\tilde{a}}_{LOS}\left(i,k\right)$ is the [(i−1)M+k]-th element of ${\tilde{a}}_{LOS}$.

### 3.3. Computational Complexity Analysis

In order to estimate the two-dimensional AOD information, if the forward-backward observing matrix $\tilde{Y}$ is also utilized, the computational cost of the typical MUSIC algorithm attains in the order of $O\left\{2GM^{2}N^{2}+M^{3}N^{3}+\delta_{1}\delta_{2}\left[MN\left(MN-K\right)+MN-K\right]\right\}$ flops, where one flop means once complex multiplications, and $\delta_{1}$ and $\delta_{2}$ are the total spectrum searching times within the search range, respectively. Besides, the reduced-dimensional MUSIC algorithm [41], which is an improved version in computational cost, attains in the order of $O\left\{2GM^{2}N^{2}+M^{3}N^{3}+\delta_{3}\left[\left(M^{2}N+M^{2}\right)\left(MN-K\right)+M^{2}\right]\right\}$ flops (based on a condition that the elevation θ and azimuth ϕ are transformed into two angles measured respectively by the LOS signal and the *x*-axis and *y*-axis). However, the proposed algorithm only attains in the order of $O\left\{2GM^{2}N^{2}+\delta_{4}M^{2}N^{2}\right\}$ flops, where $\delta_{4}$ is the total number of iterations in Algorithm 1. For an easy comparison, we ignore the small quantity of higher order and then give the ratio for the computational costs of both algorithms,

(25) $$\begin{array}{c} \frac{O\left\{2GM^{2}N^{2}+M^{3}N^{3}+\delta_{1}\delta_{2}\left[MN\left(MN-K\right)+MN-K\right]\right\}}{O\left\{2GM^{2}N^{2}+\delta_{2}M^{2}N^{2}\right\}}\approx \frac{O\left\{2G+MN+\delta_{1}\delta_{2}\right\}}{O\left\{2G+\delta_{4}\right\}}\;\;\;\;\;\;\;\;\; \end{array}$$

(26) $$\begin{array}{c} \frac{\left\{2GM^{2}N^{2}+M^{3}N^{3}+\delta_{3}\left[\left(M^{2}N+M^{2}\right)\left(MN-K\right)+M^{2}\right]\right\}}{O\left\{2GM^{2}N^{2}+\delta_{2}M^{2}N^{2}\right\}}\approx \frac{O\left\{2G+MN+\delta_{3}\left(M+\frac{M-K}{N}\right)\right\}}{O\left\{2G+\delta_{4}\right\}} \end{array}$$

Numerically, if we let the scale of antenna array M=N=10, the number of repeated codes G=20, the total number of MPC K=20, the searching step of MUSIC algorithm is set as $0.1^{\circ }$, so that it implies $\delta_{1}=900$ within the range of elevation and $\delta_{2}=1800$ within the range of azimuth. For reduced-dimensional MUSIC, $\delta_{3}=1800$. The iteration number of the proposed algorithm is smaller than 20. Therefore, the computational cost of the proposed algorithm is just approximately 1/27,000 of that of the MUSIC algorithm and is approximately 1/270 of that of reduced-dimensional MUSIC algorithm.

## 4. Distance-Weighted Positioning

We can directly utilize the multiple estimated two-dimensional AOD information to achieve the vehicle’s positioning; however, the accuracy of angle detection is vulnerable to the influence of the vehicle’s position. We herein call this problem the near-far effect, which indicates the fact that the farther the vehicle is from the RSU, the less reliable the corresponding positioning result is. For example, if one vehicle is located a large distance, it travels a little further, and the positioning will give rise to a great error even if all the conditions are the same. Furthermore, such a phenomenon will get worse in a noisy environment.

The fundamental reason behind the above phenomenon is that, when the elevation θ is large enough to approach $90^{\circ }$ and/or the azimuth ϕ approaches $0^{\circ }$ or $180^{\circ }$, the antenna array will gradually function as endfire mode. In such a mode, the effective aperture of the antenna array is greatly reduced. That is to say, there is no sufficient array aperture to guarantee a satisfactory accuracy of angle estimation.

As we know, elevation and azimuth establish a one-to-one mapping with a point on the lane plane, which means that the position has no ambiguity. Therefore, for a vehicle in a dense RSU deployment scenario, if it locates near the position that induces one RSU to work in endfire mode, correspondingly it also falls into the non-endfire mode of other RSUs. That inspired a method for reducing the adverse influence of the near-far effect, i.e., a distance based weighting strategy. It trusts the “near” positioning results more than the “far” ones. To be specific, according to the estimated two-dimensional angles, we can retrieve the distance information $D_{i}$ between the vehicle and *i*-th RSU, i.e.,

(27) $$\left\{{\hat{\theta }}_{i},{\hat{\phi }}_{i}\right\}\Rightarrow \left\{{\hat{x}}_{V,i}\left(T^{k-1}\right),{\hat{y}}_{V,i}\left(T^{k-1}\right)\right\}\overset{P_{R_{i}},z_{V}}{⟹}D_{i}$$

where $D_{i}={\left\|p_{R_{i}}-{\hat{p}}_{V,i}\left(T^{k-1}\right)\right\|}_{2}$. Without loss of generality, for K RSUs participating in cooperative positioning, we have distance information $D_{1},D_{2},\ldots ,D_{K}$, so the weighting coefficients can be determined by:

(28) $$\omega_{i}=\frac{1/D_{i}}{\sum_{i=1}^{K}1/D_{i}}.$$

Taking two RSUs for example, if $D_{1}<D_{2}$, then according to Equation (28), the weighting coefficients $\omega_{1}$ and $\omega_{2}$ are:

(29) $$\begin{array}{ccc} \omega_{1} & = & D_{2}/\left(D_{1}+D_{2}\right) \end{array}$$

(30) $$\begin{array}{ccc} \omega_{2} & = & D_{1}/\left(D_{1}+D_{2}\right) \end{array}$$

Obviously, the positioning result with small distance information will play an important role in final position determining; see Equations (3) and (4).

There exist several weighting strategies, for example weighting based on the received signal strength (RSS) (or the estimated signal-to-noise ratio (SNR)), i.e., ω∝RSS, and weighting based on the Cramér–Rao lower bound (CRB) of angle estimation, i.e., $\omega \propto \frac{1}{\mathrm{CRB}}$. Among them, the RSS falls off inversely proportional to the square of the distance between the vehicle and RSU in free-space. However, on the one hand, it is just a qualitative factor for evaluating the angle estimation and is unable to reflect the directional affect; on the other hand, although the statistical distance can be estimated from the propagation model, it is unreliable due to the random fluctuation of multipath signals. The CRB provides a bound on the covariance matrix of any unbiased estimation of angle, which includes many factors such as SNR, snapshot length *G*, and array manifold $a(\mu_{k})$ and $b(\upsilon_{k})$ [39,42]. However, there are no simple and practical methods to obtain noise variance, and also, the SNR exhibits random fluctuation [27]. In addition, the calculation of the CRB is computationally high and needs to be re-calculated when the angle information changes.

To sum up, we summarize the whole CP procedure in Table 1. Besides the basic data ${\left\{y^{\left(i\right)}\left[g\right]\right\}}_{g=1}^{G},i=1,2,\ldots ,K$ as shown in Section 3.1, the kernel of this procedures lies in Step 1, Step 2, and Step 5. Through data extension in Step 1, the accuracy of angle estimation can be improved because the Cramer–Rao lower bound is in inverse proportion to the length of observed data. In Step 2, Algorithm 1 utilizes an iterative manner to extract the signal subspace with respect to the LOS signal component rather than adopting matrix decomposition, and simultaneously, the subsequent AOD estimation in Step 3 is in a closed-form expression rather than in searching, which is computationally efficient. The final estimations of the vehicle’s positions in Step 7 actually fuse all K detected AOD information through the distance based weighting method in Step 5.

**Remark 2.**  *Looking back at the aforementioned safety distance based warning in Section 2.2, once we acquire the vehicles’ positions, the desired deceleration of vehicle $V_{2}$ can be calculated via (6) with the aid of the designed sequential task allocation in Figure 3. Due to different decelerations resulting in different driving experiences, the warning strategies can be expressed by a series of levels, for example if $a^{L-1}<a_{2d}<a^{L},L=1,2,\ldots ,L$, then the warning belongs to the L level. A large value of deceleration means a high warning level and also means a big emergency. The thresholds will be determined and evaluated by actual driving tests in the next works. Besides, the vehicle’s positioning error inevitably produces erroneous inter-vehicle distance D and further affects the desired deceleration $a_{2d}$ of vehicle $V_{2}$. In order to understand such a relationship, we try to analyze the first-order perturbation of the desired deceleration. According to (6), let the estimated distance $\hat{D}=D+\delta_{D}$, then we have:*

(31) $$\left(a_{2d}+\delta \right)\left(D+\delta_{D}-\bar{D}\right)=\frac{1}{2}{\left(\Delta v+\delta_{\Delta v}\right)}^{2}+\left(a_{1}+\delta_{a_{1}}\right)\left(D+\delta_{D}-\bar{D}\right)$$

*where $\bar{D}=D_{w}+D_{h}$ and δ, $\delta_{D}$, $\delta_{\Delta v}$, and $\delta_{a_{1}}$ denote the perturbations of the desired deceleration $a_{2d}$, the inter-vehicle distance D, the velocity difference Δv, and acceleration $a_{1}$, respectively. After a lengthy, but straightforward derivation and ignoring the second-order items, we can get:*

(32) $$\delta =\frac{a_{1}-a_{2d}}{D-\bar{D}}\delta_{D}+\frac{\Delta v}{D-\bar{D}}\delta_{\Delta v}+\delta_{a_{1}}=\rho_{1}\delta_{D}+\rho_{2}\delta_{\Delta v}+\delta_{a_{1}}$$

*This manifests that, theoretically, if other factors are correctly measured, the bias of the desired deceleration $a_{2d}$ is proportional to that of inter-vehicle distance D. In particular, based on first-order perturbation analysis, if the positioning errors in the x and y directions are independent identically distributed and of zero-mean, then $E\left\{\delta \right\}=\rho_{1}E\left\{\delta_{D}\right\}=0$. Further, the mean-squared error is $E\left\{\delta^{2}\right\}=\rho_{1}^{2}E\left\{\delta_{D}^{2}\right\}$.*

## 5. Numerical Examples

In order to demonstrate the effectiveness and advantages of the proposed AOD estimation algorithm and the localization scheme, in this section, a series of Monte Carlo numerical simulations are presented. We assume that the RSU *R* is deployed on top of a traffic light with height 6 m. The vehicle proceeds along the lane, and the lane width is set as 3.5 m. The onboard unit (OBU) antenna is deployed on the vehicle’s rooftop position with a total height of 1.8 m.

In the following simulations, the system works at carrier frequency $f_{c}=5.9$ GHz with bandwidth B=10 MHz, and the data length for positioning is fixed as G=20. For the LOS component, we adopt the dual slope model [43] to describe the path loss, i.e., the path loss $PL\left(D\right)=PL\left(D_{0}\right)+10\gamma_{1}\log_{10}\left(D/D_{0}\right)$ for $D_{0}<D\leq D_{C}$; and $PL\left(D\right)=PL\left(D_{0}\right)+10\gamma_{2}\log_{10}\left(D/D_{C}\right)+10\gamma_{1}\log_{10}\left(D_{C}/D_{0}\right)$ for $D>D_{C}$, where $PL(D_{0})$ is the signal attenuation in free-space at distance $D_{0}$. According to [43], we chose $D_{0}=10$ m, $D_{c}=80$ m, $\gamma_{1}=1.9$, and $\gamma_{1}=3.8$. Besides the LOS component, the other 20 MPCs come uniformly from any direction in angular-domain $\theta \in [0^{\circ },90^{\circ }]$ and $\phi \in [0^{\circ },180^{\circ })$; the phase and magnitude of the attenuation coefficient for each signal component are modeled as random variables with a uniform distribution in (0,2π) and (0,1), respectively. We use the Ricean κ factor to indicate the power proportion, which is defined as the ratio of the power in the LOS component to the total power in the diffused non-LOS components. Through the whole simulations, the parameter ϵ in Algorithm 1 is set as $10^{-3}$.

Simulation 1: We first consider the root mean-squared error (RMSE) performance. For comparison, we just consider one RSU with coordinate {0,0,6} participating in positioning under two cases, respectively. One is the “far” case that the vehicle locates at $\left\{\theta ,\phi \right\}=\left\{74.5^{\circ },6.7^{\circ }\right\}$, D=15.67 m; the other is the “near” case that the vehicle locates at $\left\{\theta ,\phi \right\}=\left\{39.6^{\circ },30.3^{\circ }\right\}$, D=5.45 m. The antenna array M=N=10. The noise power is given by $P_{n}=-174+10\log_{10}B=-104$ dBm for temperature T=300 K. Considering other unavoidable link loss, we let $P_{n}=-74$ dBm. According to different transmitting power, we can set a different received SNR. The antenna gains are absorbed into the SNR. Let κ=5, and the total number of Monte Carlo simulations is set as 1000.

**Figure 5:**
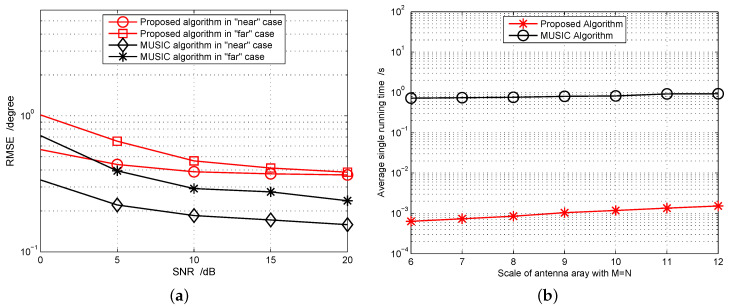
Performance evaluation for LOS angle-of-departure (AOD) estimation with different algorithms. (**a**) RMSE performance comparison; (**b**) Average single running time comparison.

Figure 5a reports the RMSE performance of the estimated LOS two-dimensional AOD. The MUSIC algorithm serves as a benchmark because it is a typical high-resolution algorithm. Correspondingly, Figure 5b gives the comparison of the average running time when executing the algorithm one time. For the MUSIC algorithm, we set the searching step as $0.1^{\circ }$ and the searching range from $\psi -5^{\circ }$ to $\psi +5^{\circ }$, ψ∈{θ,ϕ}. From the simulation results, we can make two conclusions. First, for both cases, the RMSE performance of the proposed algorithm is slightly inferior to the MUSIC algorithm; however, the much lower computational complexity makes it a better alternative to the exhaustive searching algorithm. Second, at the same SNR level, the “far” position manifests inferior error performance to the “near” one, which proves the near-far effect in angle based positioning.

**Figure 6:**
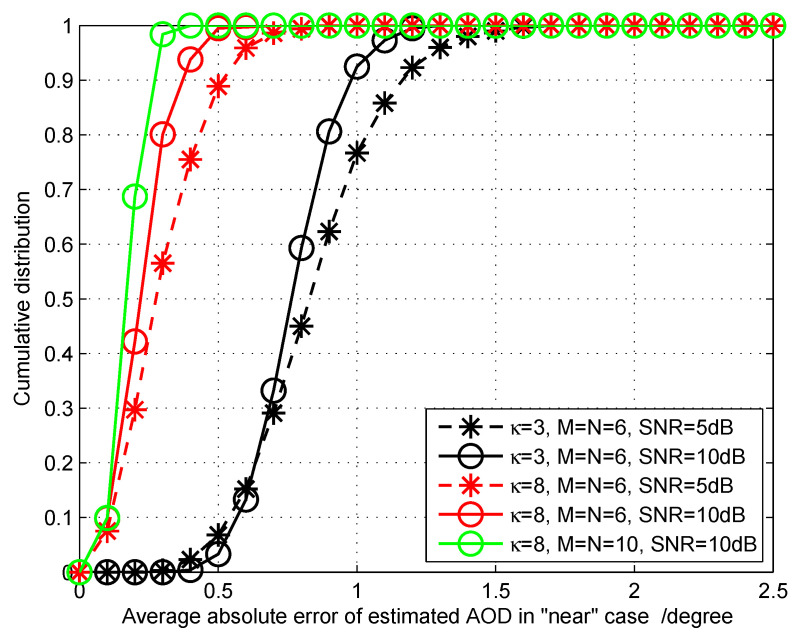
The cumulative distribution of the average absolute angle estimation error under different conditions.

Simulation 2: We then consider the cumulative distribution of the average absolute error for LOS AOD estimation provided by the proposed algorithm. This evaluation criterion is defined by $P_{\zeta }\left(\theta_{1},\phi_{1}\right)=P\left\{\left[|{\hat{\theta }}_{1}-\theta_{1}|+|{\hat{\phi }}_{1}-\phi_{1}|\right]/2\leq \zeta \right\}$, where ζ denotes a series of allowed angle scales. We choose ϵ from $0^{\circ }$ to $2.5^{\circ }$ with step $0.1^{\circ }$. The purpose of this simulation is to examine the error level under different conditions. The simulation results is shown in Figure 6. We can conclude that the AOD estimation accuracy can be improved with the increase of the κ factor, the scale of the antenna array, and the SNR. For example, at κ=3, M=N=6, and SNR=10 dB, the error cumulative distribution shows $P_{\zeta }\left(\theta_{1},\phi_{1}\right){|}_{\zeta =1.3^{\circ }}=1$, and it turns to $P_{\zeta }\left(\theta_{1},\phi_{1}\right){|}_{\zeta =0.5^{\circ }}=1$ at κ=8, M=N=10, and SNR=10 dB, which illustrates that the error values are strongly converging.

Simulation 3: We now evaluate the positioning performance. For convenience, we assume that there are two RSUs locating at {0,0,6} m and {12,0,6} m, respectively. One vehicle travels along the middle line of the lane and passes five points where the CP is launched. The coordinates of these points and the corresponding AOD and distance information are listed in Table 2. The transmitting power is set as 10 dBm. The Ricean κ=3. The path loss model is the same as previous simulations.

**Figure 7:**
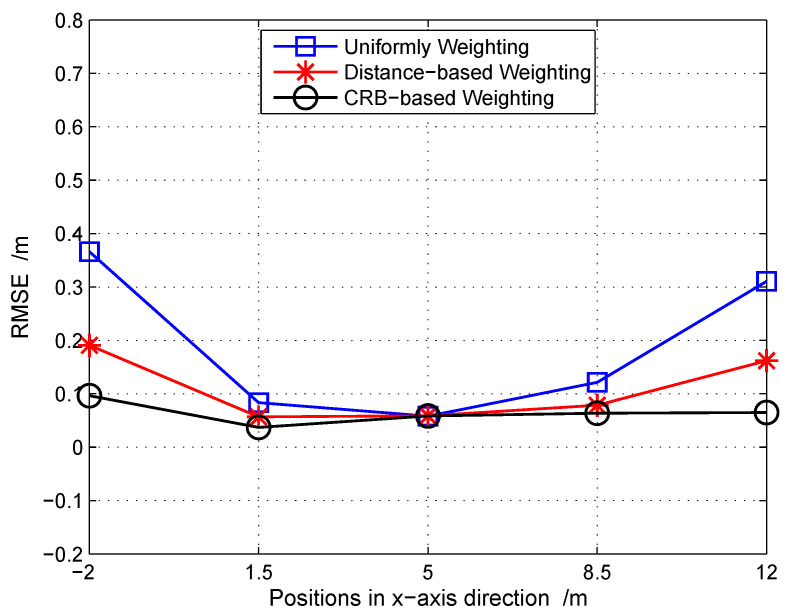
The positioning RMSE comparison for different weighting strategies.

Besides the proposed distance based weighting, we also compare two different strategies, i.e., the uniform weighting and the CRB based weighting. Figure 7 gives the positioning RMSE of all five points. As we can see, the CP with distance based weighting performs better than the uniform weighting and is slightly inferior to the CRB based weighting. It is worth mentioning that the CRB based weighting stems from the complicated CRB expression [39]; although it gives the smallest error in positioning, the fast and accurate calculation is impractical because, on the one hand, the array manifold of all MPCs and noise variance should be known and, on the other hand, the computational burden is heavy. Oppositely, the proposed one makes a better trade-off between the positioning accuracy and the computational complexity.

## 6. Conclusions

We designed a basic framework of a joint cooperative positioning and warning system from the perspective of angle-awareness. In this framework, the cooperative positioning model based on state representation, the warning mechanism based on safety distance, and the sequential task allocation were discussed. Besides, in order to reduce the computational complexity of angle-awareness and improve the cooperative positioning accuracy, we proposed a truncated signal-subspace based algorithm for AOD estimation and a distance based weighting strategy for position estimation, respectively. Compared with the exhaustive searching based algorithms such as two-dimensional MUSIC, the proposed one maintains acceptable performance and decreases the computational complexity. Besides, the proposed distance based weighting method also achieves a similar level of positioning accuracy as the theoretical CRB based weighting, which is more practical. Therefore, both proposed methods can be used as better alternatives in a practical positioning and warning system. Actually, there exist some important issues that need to be considered; therefore, future works will focus on the optimization of the deployment of RSUs, the real-time high-accuracy trajectory tracking based on Kalman filtering, and the fusion of supplementary information such as a camera or LIDAR. 

## Figures and Tables

**Table 1:** The vehicle’s position estimation procedures.

**Given**	A series of received data from K RSUs, ${\left\{y^{\left(i\right)}\left[g\right]\right\}}_{g=1}^{G},i=1,2,\ldots ,K$.
**Step 1.**	(**Data Extension**) Construct ${\tilde{Y}}^{\left(i\right)}$ via (17).
**Step 2.**	(**Subspace Extraction**) Run Algorithm 1 to obtain ${\tilde{a}}_{LOS}^{\left(i\right)}$.
**Step 3.**	(**Parameters’ Estimation**) Estimate $\mu_{1}^{\left(i\right)}$ and $\upsilon_{1}^{\left(i\right)}$ according to (23) and (24).
**Step 4.**	(**Parameters’ Conversion**) Convert ${\hat{\mu }}_{1}^{\left(i\right)}$ and ${\hat{\upsilon }}_{1}^{\left(i\right)}$ into $\theta_{1}^{\left(i\right)}$ and $\phi_{1}^{\left(i\right)}$ by (9).
**Step 5.**	(**Weighting Determination**) Determine the weighting coefficients by (28).
**Step 6.**	(Data Traversal) Repeat **Step 2**∼**Step 5** until all the data of K RSUs are processed.
**Step 7.**	(**Position Calculation**) Calculate the vehicle’s positions during $T_{TR}$ by (3) and (4) and calculate other positions by (2).

**Table 2:** Parameter arrangement for positions to be located.

Para.	$x_{V}$	$y_{V}$	*D*	θ	ϕ
Ref. No.					
RSU-1 @ {0,0,6} m	−2 m	1.75 m	5.0 m	$32.3^{\circ }$	$131.2^{\circ }$
	1.5 m	1.75 m	4.79 m	$28.7^{\circ }$	$49.4^{\circ }$
	5 m	1.75 m	6.76 m	$51.6^{\circ }$	$19.3^{\circ }$
	8.5 m	1.75 m	9.65 m	$64.2^{\circ }$	$11.6^{\circ }$
	12 m	1.75 m	12.8 m	$70.9^{\circ }$	$8.3^{\circ }$
RSU-2 @ {12,0,6} m	−2 m	1.75 m	14.62 m	$73.4^{\circ }$	$172.9^{\circ }$
	1.5 m	1.75 m	11.43 m	$68.5^{\circ }$	$170.5^{\circ }$
	5 m	1.75 m	8.35 m	$59.8^{\circ }$	$166^{\circ }$
	8.5 m	1.75 m	5.74 m	$43^{\circ }$	$153.4^{\circ }$
	12 m	1.75 m	4.55 m	$22.6^{\circ }$	$90^{\circ }$
